# Computed tomography in traumatic orbital emergencies: a pictorial essay—imaging findings, tips, and report flowchart

**DOI:** 10.1186/s13244-021-01142-y

**Published:** 2022-01-12

**Authors:** Michaela Cellina, Maurizio Cè, Sara Marziali, Giovanni Irmici, Daniele Gibelli, Giancarlo Oliva, Gianpaolo Carrafiello

**Affiliations:** 1 Radiology Department, Fatebenefratelli Hospital, ASST Fatebenefratelli Sacco, Piazza Principessa Clotilde 3, 20121 Milan, Italy; 2 Scuola di Specializzazione in Radiodiagnostica, Università degli Studi di Milano, Via Festa del Perdono, 7, 20122 Milan, MI Italy; 3 Dipartimento Di Scienze Biomediche Per La Salute, Università Degli Studi Di Milano, Via Mangiagalli 31, 20133 Milan, Italy; 4 Radiology Department, Policlinico di Milano Ospedale Maggiore, Fondazione IRCCS Ca’ Granda, Via Francesco Sforza, 35, 20122 Milan, MI Italy

**Keywords:** Tomography (X-ray computed), Orbital emergencies, Orbital fractures, Orbital foreign bodies, Orbital trauma

## Abstract

Computed tomography (CT) is considered the gold standard technique for the assessment of trauma patients with suspected involvement of the eye and orbit. These traumas can result in dramatic consequences to visual function, ocular motility, and aesthetics. CT is a quick and widely available imaging modality, which provides a detailed evaluation of the orbital bony and soft tissue structures, an accurate assessment of the globes, and is used to guide the patients’ treatment planning. For a timely and accurate diagnosis, radiologists should be aware of fracture patterns and possible associated complications, ocular detachments and hemorrhages, and different appearances of intraorbital foreign bodies. This educational review aims to describe all post-traumatic orbital abnormalities that can be identified on CT, providing a list of tips and a diagnostic flowchart to help radiologists deal with this complex condition.

## Key points

- Computed Tomography (CT) is the gold standard for orbital trauma assessment.
- CT enables evaluation of orbital fractures, soft tissue injuries, foreign bodies.
- CT multiplanar reconstructions help surgical planning of orbital fractures.
- CT allows the visualization of the ocular globe content and structures.

## Introduction

Craniofacial trauma represents a public health issue of pivotal relevance due to its frequency and the high number of accesses to the Emergency Departments. These traumas may be often associated with damage of the eye and orbit, resulting in significant functional impairment if not timely diagnosed and properly managed.

In the United States, eye injuries account for 3% of all Emergency Departments accesses, with more than 2.5 million new eye injuries each year [1–3].

In a traumatic setting, clinical evaluation can be hindered by soft tissue swelling or damage and patients’ general conditions. In these cases, CT represents the gold standard for investigating suspected orbital fractures [4] and identifying foreign bodies, due to the excellent bone and soft-tissue visualization [5]. Thanks to the possibility of multiplanar and tridimensional reconstructions, CT is also the primary imaging modality for surgical planning of fracture reconstructions and treatment management [6].

Radiologists have a key role in diagnosing, assessing bone and orbital soft tissue damage, as well as identifying potential complications. However, Radiological Departments do not always have neuroradiologists dedicated to reporting emergencies on duty, and the general radiologists who are faced with imaging of orbital emergencies may find the topic quite challenging.

This article aims to display and describe the CT findings of traumatic orbital emergencies collected in a 24/7 Ophthalmological Emergency Department in the period from January 2019 to June 2021, and provide a structured flowchart to help the radiologist recognize the most frequent fracture patterns, assess and describe orbital, soft tissue and globe damage.

This observational retrospective study follows international laws and guidelines (Helsinki Declaration), with experimentation Register Number 2018/ST/191. Patients provided written consent for the use of their anonymized images.

### Hints of anatomy

Even if the orbit is a relatively small pyramidal space, it has a complex anatomy and includes critical structures.

The orbital bony structure is made up of a floor, a roof, a medial, and a lateral wall. The orbital roof is the floor of the frontal sinus creating a division from the anterior cranial fossa; most of it corresponds to the orbital surface of the frontal bone. The lesser wing of the sphenoid bone contributes to a minor portion of the roof at the orbital apex [7]. The orbital floor represents the roof of the maxillary sinus, and it is mainly formed by the maxilla orbital process. A portion of the zygomatic bone constitutes the anterior side, while a little contribution of the palatine bone creates the posterior side [8].

From front to back, the maxillary bone, the lacrimal bone, the ethmoid bone, and the body of the sphenoid bone together contribute to the medial wall, which divides the orbital content from the ethmoidal cells [9].

The lateral wall is made up of the zygomatic bone anteriorly and the greater wing of the sphenoid bone posteriorly [9].

The globe is placed in the anterior part of the orbit, and its content is bordered by three layers: the sclera and cornea form the outermost layer, the uveal tract, with the ciliary body anteriorly and the choroid posteriorly, forms the middle layer, and the retina forms the inner layer. These layers are intertwined and cannot be distinguished on CT imaging in a healthy eye; therefore they are visualized as a single layer [10].

The lens is an avascular proteinaceous component that divides the globe into an anterior segment filled with aqueous humor, and a posterior segment, containing the vitreous humor, accounting for 2/3rds of the total globe volume [10].

The lens is attached to the sclera through the zonular fibers and is responsible for the accommodation. It contributes around 1/3rd of the total optical power [10].

The anterior segment is further divided by the iris into an anterior and posterior chamber.

The extraocular muscles are seven and together form a conical shape. Six of the extraocular muscles control the movement of the eye, while the remaining muscle, the levator palpebrae, is responsible for superior eyelid elevation [11]. The four rectus muscles—medial, lateral, inferior, and superior—originate from a common tendinous ring, called the annulus of Zinn, in the back of the orbit, and attach directly to the front half of the eye. The two oblique muscles—superior and inferior—have an oblique course and attach to the posterior surface of the sclera.

The levator palpebrae superioris originates from the lesser wing of the sphenoid bone and attaches to the superior tarsal plate of the upper eyelid [11].

The optic nerve can be divided into an intracranial and extracranial tract [12]. It is composed of the convergence of axons from the retinal ganglion cells; it runs in the center of the orbit and exits through the optic canal in the sphenoid bone and is surrounded by the optic nerve sheath, which is an extension of the dura mater.

The ophthalmic artery originates from the internal carotid at the medial section of the anterior clinoid process and runs anteriorly, going through the optic canal, inferolateral to the optic nerve. All of the orbital structures are supplied by its branches [12]. The superior ophthalmic vein is located between the superior rectus muscle above, and the ophthalmic artery and optic nerve below; it exits the orbit through the superior orbital fissure [12].

### Orbital fractures

Orbital fractures are typically the result of middle third facial traumas with the application of forces that overcome the resistance of the orbital cavity bones [13].

They can be single-wall or multi-wall, with the latter being the more prevalent. Moreover, there are complex facial fracture patterns with orbital involvement: the zygomaticomaxillary complex fracture, nasoorbitoethmoid fractures, and Le Fort fractures.

Orbital fractures can be classified as blow-out and blow-in [14]. Blowout fractures (BOFs) are orbital wall fractures with orbital rim sparing that are caused by direct trauma to the orbit resulting in a sudden increase in intraorbital pressure, with bone fragments displaced caudally into the maxillary sinus. Blow-in fractures determine a reduction of the intraorbital space due to the internal displacement of bony fragments and are usually associated with proptosis [13].

The most common clinical signs of orbital fractures are periorbital edema, periorbital ecchymosis, subconjunctival hemorrhage, subcutaneous emphysema with crepitus, periorbital contusions, hematomas, soft tissue injuries involving the eyelids, and deficits of the infra-orbital tract of the optic nerve [15].

BOFs are usually associated with injuries to surrounding soft tissues and orbital cavity contents. They may cause entrapment or herniation of orbital fat and extraocular muscles, resulting in limited eye movements and/or enophthalmos due to intra-orbital volume reduction [15].

The inferior orbital wall is the most commonly involved site, followed by the medial orbital wall.

The indications to orbital repair are still unclear; absolute indications to surgical repair are as follows: a clinically evident enophthalmos and/or hypophthalmos, a severe restriction of ocular motility with CT evidence of muscle entrapment or incarceration of periorbital soft tissue, a “white eye blowout” fracture in children or young adults with strict restriction of ocular motility and vagal symptoms, compartment syndromes (superior orbital fissure and orbital apex syndromes) needing urgent surgical decompression. Relative indications involve the presence of orbital floor defects > 50% of the orbital floor or an affected area of > 1 cm^2^, and persistent diplopia due to entrapment or fibrosis of the extra orbital muscles [16].

**Fig. 1:**
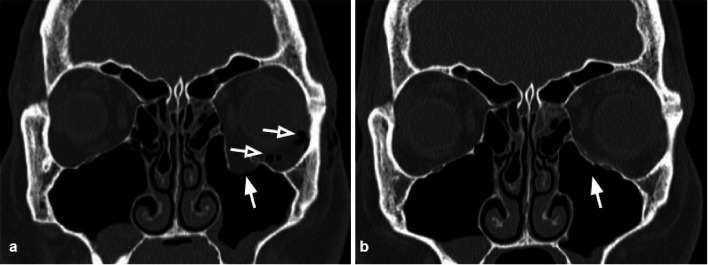
Coronal CT reconstructions. **a** Blow-out fracture of the left orbital floor (arrow) with herniation of the intraorbital fat into the maxillary sinus. Presence of intraorbital emphysema, an indirect sign of fracture (empty arrows). **b** Coronal reconstruction of the follow-up CT 2 months after the orbital wall reconstruction through the position of an absorbable mesh. The integrity of the orbital floor is restored (white arrow); the soft tissue herniation is no longer present

Surgical management consists of the removal of displaced bone fragments and internal fixation of the fracture through the positioning of mesh materials to restore the original orbital volume and morphology (Fig. 1) [16].

### Orbital floor fractures

**Fig. 2:**
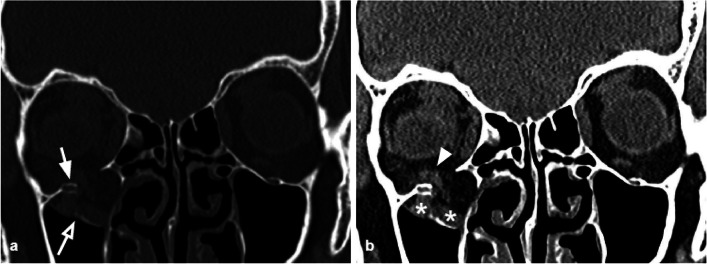
Coronal CT reconstruction with bone (**a**) and soft tissues (**b**) algorithms of an isolated orbital floor fracture*.* **a** Right orbital floor fracture with involvement of the medial aspect of the infraorbital groove (white arrow) and dislocation of a bony fragment into the maxillary sinus (empty arrow). **b** Mild swelling of the right inferior rectus muscle (arrowhead) partially herniated into the bony defect; huge herniation of the intraorbital fat through the fracture gap; within the herniated soft tissue, hematomas are recognizable (asterisks)

**Fig. 3:**
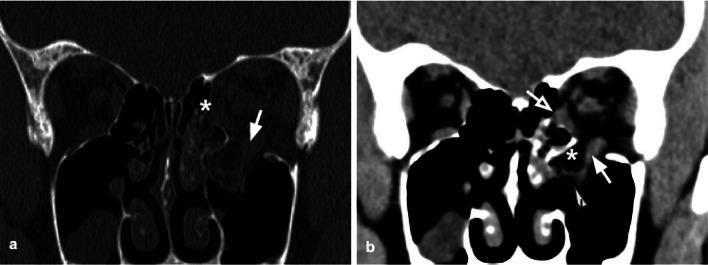
Coronal CT reconstruction with bone (**a**) and soft tissues (**b**) algorithms of an orbital floor fracture associated with a lamina papyracea fracture. **a** Left orbital floor fracture with huge bone defect (arrow), associated with medial wall fracture (white asterisk). **b** Herniation of the intraorbital soft tissues into the maxillary sinus (white asterisk). The inferior rectus muscle is dysmorphic, elongated, and also partially herniated caudally into the sinus (arrow). In the lower portion of the herniated tissue, a small hematoma is present (arrowhead). The medial rectus muscle (white asterisk) is swollen and partially entrapped in the fracture of the lamina papyracea (empty arrow)

The orbital floor BOF is the most common fracture and can occur alone (Fig. 2) or in combination with other facial fractures (approximately 50% of cases it is associated with the medial wall fracture). It is often characterized by bone fragments displaced into the maxillary sinus [17] (Figs. 2, 3).

The most prevalent cause of injury is direct anteroposterior blunt trauma to the globe and orbital boundaries. The potential mechanism for this sort of fracture has been proposed as hydraulic energy and buckling of the floor from impact to the inferior orbital edge [14].

**Fig. 4:**
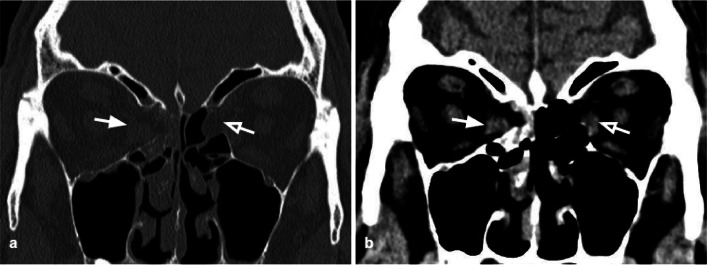
Coronal CT reconstruction with bone (**a**) and soft tissues (**b**) algorithms in a patient with a current fracture of the right orbital medial wall (white arrow) and a previous fracture of the contralateral orbital medial wall (empty arrow). **a** Huge bone defect of the right lamina papyracea (white asterisk). On the left side, discontinuation of the lamina papyracea caused by a previous fracture is visible, as well as the herniation of the intraorbital fat tissue into the ethmoidal cells (empty arrow). **b** The medial rectus is thickened and partially displaced through the bone defect (arrow). The left medial rectus muscle shows regular site and morphology (empty arrow)

Orbital floor fractures can be complicated by the intraorbital fat herniation towards the maxillary sinus, and entrapment of the inferior rectus muscle [16] (Figs. 3, 4). The extent of the transverse fracture and the presence of soft tissue herniation on CT can help predict the development of chronic diplopia, and the need for surgical repair [18].

A particular type of orbital floor BOFs is represented by the trapdoor fracture, which usually occurs in children and toddlers due to the distensibility of immature facial bones [19]. In this type of fracture, the inferior rectus muscle and/or intraorbital fat prolapse through the fracture defect into the underlying maxillary sinus, and the fracture bony fragment springs back into place, acting like a trapdoor. These patients present with limitations in the supraduction of the globe, resulting in diplopia, and often show signs of oculocardiac reflex: bradycardia, nausea, and vomiting [20]. This fracture is also known as “white-eye blow-out fracture”. On coronal CT reconstructions, the inferior rectus muscle or the intraorbital fat herniates through a non-displaced inferior orbital wall fracture.

Tips: assess the presence of the herniation of the intraorbital fat and/or inferior rectus muscle through the bone defect.

Remember to check the integrity of the inferomedial orbital strut, a bony thickening that extends from the maxillary to the ethmoid bone, whose anterior part represents an attachment site for the suspensory ligaments. The fracture involvement of the inferomedial orbital strut increases the difficulty of surgical repair, with the risk of globe malposition and eye movement impairment.

### Medial wall fractures

The medial wall is the second most common site of orbital fractures, occurring through the lamina papyracea. It can be isolated or associated with other wall fractures and caused by anteroposterior traumas directed to the globe and the orbital borders. The most common type of presentation is an association of the medial wall and floor (36%) fractures or medial wall, floor, and zygomatic complex (28%) fractures [21].

Non-displaced lamina papyracea fractures can be difficult to detect: the presence of orbital emphysema can be considered its radiological indirect sign [21].

The radiologist should accurately assess the orbit medial anatomical structures, such as medial canthal tendon, lacrimal apparatus, medial rectus, and superior oblique muscles [22].

Indications for surgery include the restriction of ocular motility due to entrapment of the medial rectus muscle, diplopia, and clinically significant enophthalmos. Enophthalmos is a rare occurrence in isolated medial wall fractures but may be present in the case of associated floor fractures [23].

**Fig. 5:**
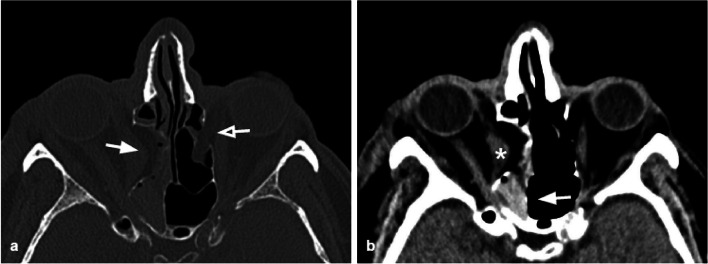
Axial CT acquisition with bone (**a**) and soft tissues (**b**) algorithms in a patient with a current fracture of the right orbital medial wall (white arrow) and a previous fracture of the contralateral orbital medial wall (empty arrow). **a** Extensive discontinuation of the right lamina papyracea (white arrow). **b** The medial rectus muscle is swollen, displaced medially, and partially entrapped into the fracture (asterisk); presence of hemosinus, visible as a right posterior ethmoidal cell occupied by hyperdense material (blood) (arrow)

Tips: assess the presence of herniation of the intraorbital fat through the bone defect and/or medial rectus muscle entrapment (Figs. 4, 5).

### Orbital roof fractures

The superior orbital wall, also known as the orbital roof, separates the anterior cranial fossa from the intraorbital content. Fractures through the orbital roof are typically the result of a high-energy facial trauma to the forehead or superior orbital rim, usually associated with intracranial damages and involvement of the orbital rim [24]. Isolated fractures of the orbital roof are uncommon, most frequently observed in children, as the frontal sinus is the last paranasal sinus to develop [25].

The fracture fragments may be displaced superiorly (BOFs), or inferiorly (“blow-in” fracture), or non-displaced. Orbital roof fractures are usually treated conservatively; however, decreased ocular motility may occur in case of entrapment of the superior rectus and superior oblique muscles [26]. Furthermore, orbital roof fractures may also determine intracranial complications connected to the anatomical relationship with the anterior cranial fossa: dural tears, cerebrospinal fluid leak, pneumocephalus, diffuse cerebral edema, meningitis, and cerebral contusion. Their resolution may require both intracranial and extracranial approaches [27–29].

**Fig. 6:**
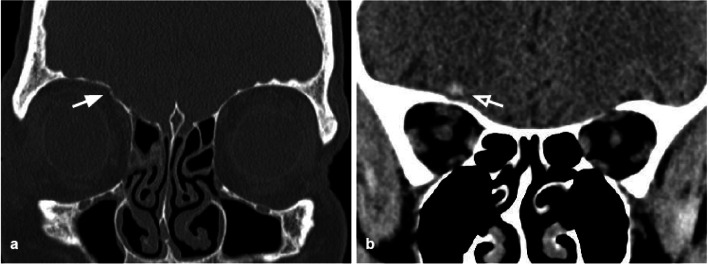
Coronal CT reconstruction with bone (**a**) and soft tissues (**b**) algorithms of an orbital bone fracture. Non-displaced fracture of the orbital roof (white arrow) (**a**), associated with intracranial complications: in figure **b**, a small frontal intraparenchymal brain hematoma is visible as a focal hyperdensity (empty arrow)

Tips: look for entrapment of the superior rectus and superior oblique muscles, and possible intracranial complications (Fig. 6).

### Lateral orbital wall fractures

**Fig. 7:**
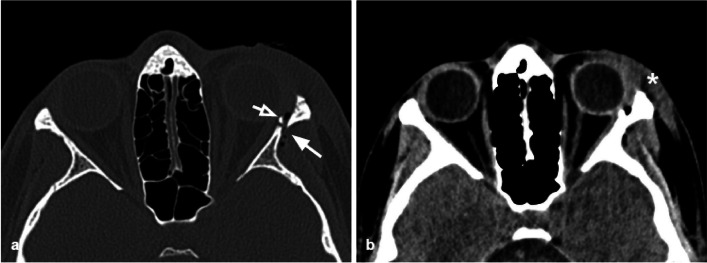
Axial CT acquisition with bone (**a**) and soft tissues (**b**) algorithms of a pure fracture of the left lateral orbital wall (white arrow). Slightly displaced fracture of the lateral orbital wall with adjacent intraorbital small air bubbles. A small bony fragment (empty arrow) presents contact with the lateral rectus muscles, that preserves regular morphology and thickness. Hematoma of the subcutaneous soft tissue (asterisk)

The lateral orbital wall is the hardest of all the orbital walls; therefore pure lateral wall fractures are extremely uncommon (Fig. 7); however, the lateral wall can develop diastasis, displacement, and comminution, in the case of zygomatic-orbital complex fractures, and high-energy traumas [30]. Untreated lateral wall fractures can favor the development of enophthalmos.

### Zygomaticomaxillary complex fractures

**Fig. 8:**
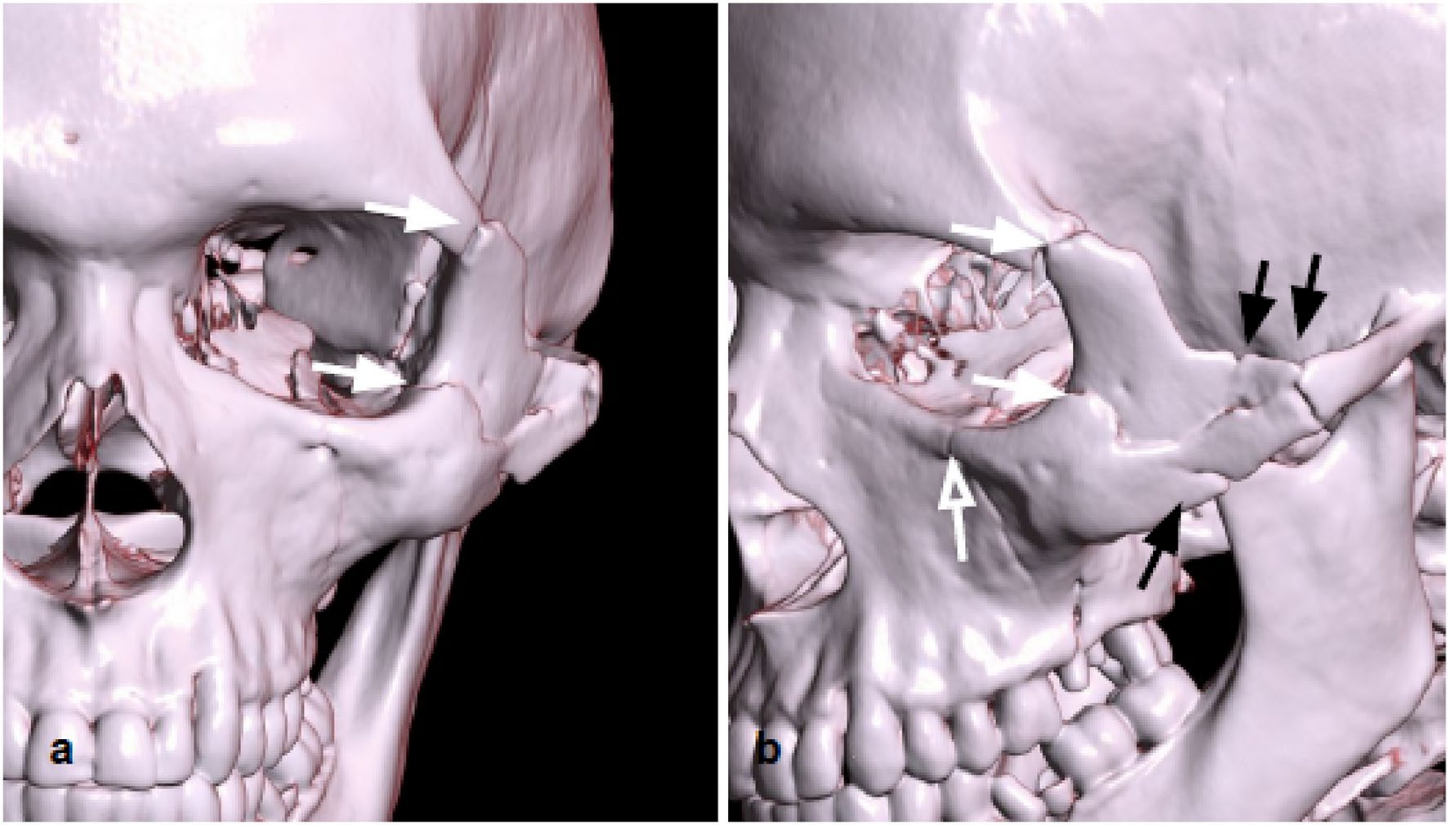
3D reconstructions of a left zygomaticomaxillary complex fractures. Fractures of the lateral orbital wall (white arrow), of the orbital floor (empty arrow), multifocal fracture of the zygomatic arch (black arrows)

Zygomaticomaxillary complex fractures are the most common facial fractures with orbital involvement, usually caused by a high-energy direct traumatic blow to the malar eminence, resulting in fractures of the lateral and inferior orbital borders and extension into the anterior wall of the maxillary sinus, zygomatic arch, and internal lateral orbital wall (Fig. 8) [30].

The zygomatic maxillary complex is made up of four bony articulations. Superiorly, the zygoma frontal process articulates with the frontal bone; medially, the zygomatic bone articulates with the maxillary bone. The zygomatic bone articulates with the temporal bone zygomatic process on the lateral side, contributing to a part of the zygomatic arch. Posteriorly, it articulates with the sphenoid greater wing, to form the orbit internal lateral wall [24]. Fracture of the four articulations of the complex can be characterized on CT by evidence of fractures of the lateral and inferior orbital borders, combined with the involvement of the anterior wall of the maxillary sinus, zygomatic arch, and internal lateral orbital wall [30].

Displaced fractures with considerable lateral angulation of the orbital internal lateral wall or simultaneous orbital floor blowout fractures can cause enophthalmos, which requires urgent surgical correction and intraorbital reconstruction [31]. CT is crucial in determining the degree of comminution and angulation of the internal lateral orbital wall, as well as the presence of a concurrent floor fracture: in this case, the presence of inferior rectus muscle entrapment should be assessed [32].

**Fig. 9:**
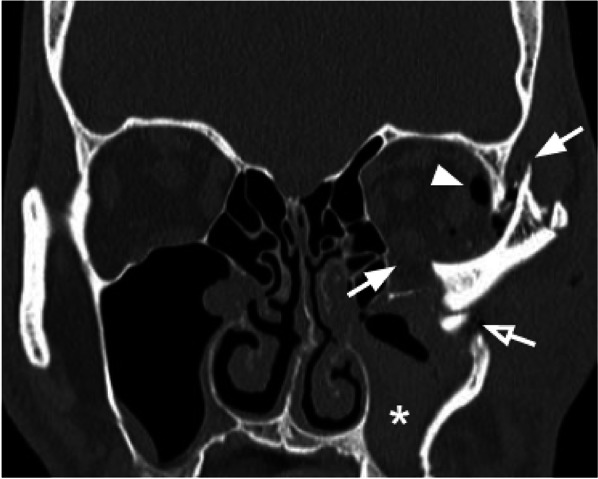
Coronal reconstruction with the bone algorithm of a left lateral orbito-zygomatic fracture. Coexistence of fracture of the orbital floor and the lateral wall (white arrows), fracture of the lateral wall of the left maxillary sinus (empty arrow), and fracture of the zygomatic arch, with medial intraorbital displacement. Presence of intraorbital emphysema (arrowhead) and hemosinus (asterisk)

Tips: check for the comminution and the position of the bone fragments of the internal lateral orbital wall (Fig. 9).

### Naso-orbitoethmoidal complex fractures

**Fig. 10:**
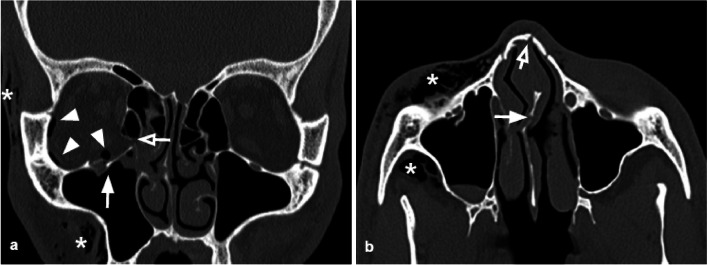
Coronal reconstruction (**a**) and axial CT acquisition (**b**) of a Naso-orbitoethmoidal Complex Fracture. Coexistence of a fracture of the right orbital floor with the involvement of the infraorbital groove (white arrow, figure **a**) and of a fracture of the lower third of the right lamina papyracea (empty arrow, figure **a**), and of a fracture of the nasal septum (arrow) and of the left nasal bone (empty arrow) in figure (**b**). Evidence of intraorbital (white arrowheads, figure **a**) and soft tissue (white asterisks, figure **a**, **b**) emphysema

Naso-orbitoethmoidal complex fractures (Fig. 10) are caused by a posteriorly oriented high-impact force applied to the nasoethmoidal region, with transmission through the nasal cavity, medial orbital wall, and ethmoid sinuses, resulting in the fractures of the ethmoid sinuses and medial orbital wall, and usually associated with the fractures of the nasal bone and septum. This type of fracture is frequently characterized by a BOF of the medial orbital wall, with intraorbital volume loss, and is often associated with complications such as telecanthus (increased distance between the medial eyes canthi) due to medial canthal tendon injury,

nasolacrimal duct lesion with impaired tear drainage [33] and cerebrospinal fluid rhinorrhea caused by the cribriform plate fracture with a dural tear, which is considered one of the most difficult fractures patterns for surgery [34].

According to Markowitz and Manson's classification, the naso-orbitoethmoidal complex fractures can be divided into three types: Type I is a single-segment central fragment with no involvement of the medial canthal tendon attachment; type II is a comminuted central fragment with fractures external to the medial canthal tendon insertion, and type III is a medial canthal tendon avulsion with a comminuted lacrimal fossa fracture [33]. Injuries can be further classified as unilateral and bilateral and according to the extension into other anatomic areas [33]. Although the medial canthal tendon is not visible on CT, the degree of comminution of the medial orbital wall at its expected attachment in the lacrimal fossa can help guide surgical repair planning [34]. Up to 20% of patients affected by this type of fracture present with nasolacrimal duct involvement that can evolve in impaired tear drainage if not timely treated [24].

Tips: assess the fracture degree at the attachment of the medial canthal tendon. Look for nasolacrimal duct injuries.

### Le Fort complex fractures

**Fig. 11:**
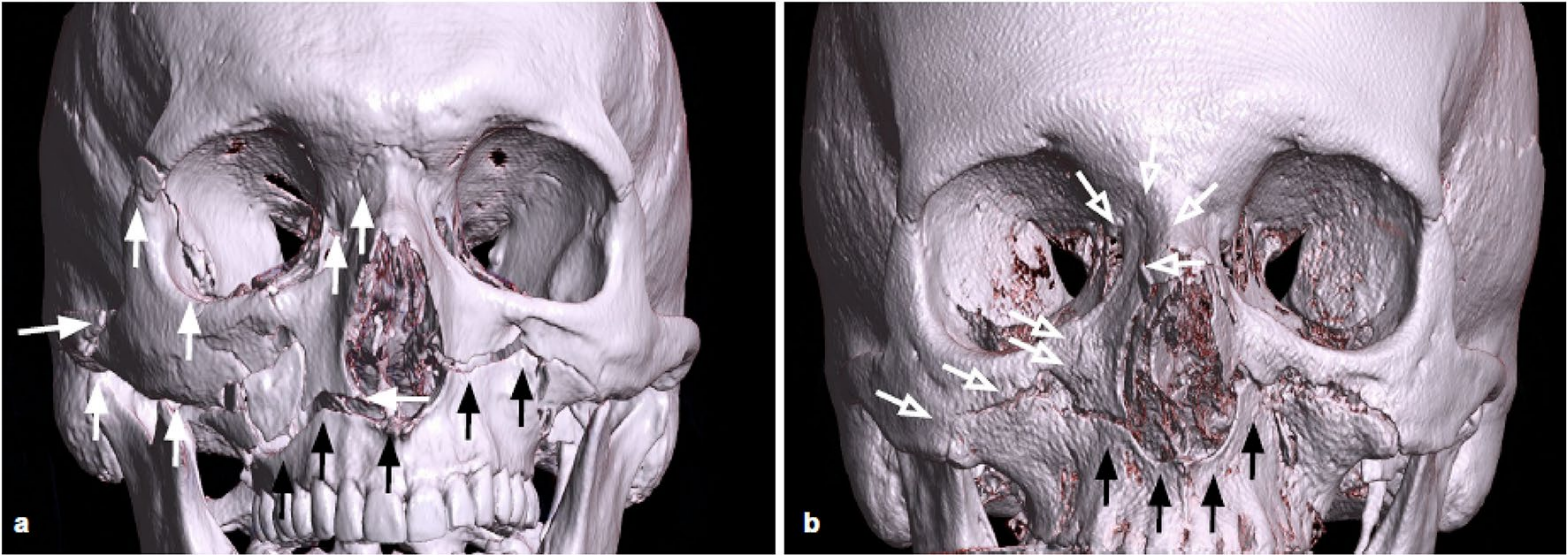
3D reconstructions of Le Fort Fractures. Both patients presented with Le Fort I fracture (black arrows), with horizontal maxillary fracture which passes through the alveolar ridge, lateral nose, and inferior maxillary sinuses wall, separating the teeth from the upper face. Patient **a** also showed a right Le Fort III fracture (white arrows), also known as craniofacial disjunction, consisting of fractures involving the nasofrontal suture, maxillo-frontal suture, orbital wall, and zygomatic arch/zygomaticofrontal suture. Patient **b** showed a Le Fort II (empty arrows), with a pyramid shape, with a fracture line involving the nasofrontal junction, the alveolar ridge, and the lateral wall of the right maxillary sinus

Fractures involving complete separation of all or a portion of the maxilla from the skull base are commonly described according to the classification proposed by French surgeon Rene Le Fort in 1901 [35]. The pterygomaxillary junction disjunction is the common characteristic shared by all of the three patterns. The Le Fort type I fracture complex does not involve the orbit, but the fracture of the inferior portions of both the lateral and medial maxillary buttresses. The Le Fort II pattern involves fractures through the zygomaticomaxillary and fronto-maxillary sutures (Fig. 11).

The Le Fort III pattern involves complete craniofacial dissociation, with fracture of the orbital medial and lateral walls and the zygomatic arch [34] (Fig. 11).

The Le Fort II, also known as the pyramidal fracture, involves the inferior and medial orbital walls and results in a pyramid-shaped fracture of the central midface that may detach from the remaining lateral face and skull base, whereas type III involves the lateral orbital wall and zygomatic arch causing craniofacial separation [30].

These fracture patterns may involve both sides of the midface symmetrically or asymmetrically [30].

Tips: the use of all multiplanar and 3D reconstructions can help the characterization of Le Fort fractures.

### Orbital apex fractures

The orbital apex corresponds to the posterior portion of the orbit and is one of the most critical regions when involved in fractures. Fractures of the orbital apex can extend through the optic canal or superior orbital fissure, resulting in damage or impingement to the neurovascular structures that run through these bony canals.

Two clinical syndromes may complicate orbital apex fractures: the superior orbital fissure syndrome and the orbital apex syndrome. The superior orbital fissure syndrome is the result of injury to cranial nerves III, IV, V1, and VI, that cross the superior orbital fissure, and present with ophthalmoplegia, diplopia, and ptosis. The combination with the damage to the optic nerve at the orbital apex results also in unilateral vision loss: this condition is known as orbital apex syndrome. Even if MRI remains the gold standard for optic nerve assessment [36], CT analysis should include a careful evaluation of retrobulbar hematoma, bone fragments causing the nerve impingement, and appearance of the intracanalicular tract of the optic nerve [30]. The decrease in visual acuity due to the orbital apex compression is considered a surgical emergency [37].

Tips: look for the intracanalicular tract of the optic nerve, and check for possible compression by bony fragments or retrobulbar hematoma.

### Traumatic optic neuropathy (TON)

It consists of an acute injury to the optic nerve, resulting from direct trauma to the optic nerve or an indirect insult due to diffuse axonal injury from blunt head trauma.

**Fig. 12:**
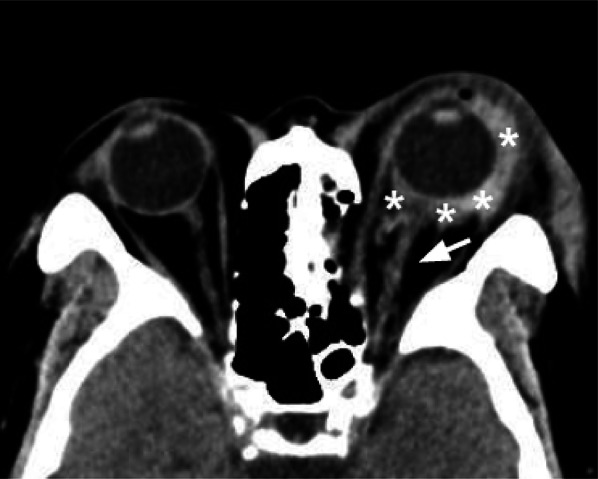
Axial CT acquisition reconstructed with soft tissue algorithm in an orbital blunt trauma with fracture of the left lamina papyracea, in a patient who complained left visual loss. The left optic nerve is stretched, mildly swollen, and hyperdense (white arrow). These findings are suggestive for post-traumatic optic neuropathy. The globe is surrounded by a hyperdense hematoma (asterisks), extended in the retro-orbital fat tissue next to the optic nerve

CT plays a limited role in diagnosing TON. It can demonstrate an optic canal fracture and, in a few cases, CT can visualize optic nerve stretching, swelling, and densitometric changes [38] (Fig. 12).

CT can help the management of suspected TON: if the optic nerve is partially or completely avulsed, aggressive measures are not indicated. In the case of visual loss, with an intact optic nerve, high-dose intravenous corticosteroids may be considered as a treatment. Surgical exploration may be indicated in nerve impingement, especially if a bone fragment or other material is compressing the optic nerve.

Tips: in patients complaining of sudden post-traumatic decreased visual acuity, the radiologist should pay attention to the orbit apex to exclude the presence of fractures.

### Anterior chamber hemorrhage (hyphema)

**Fig. 13:**
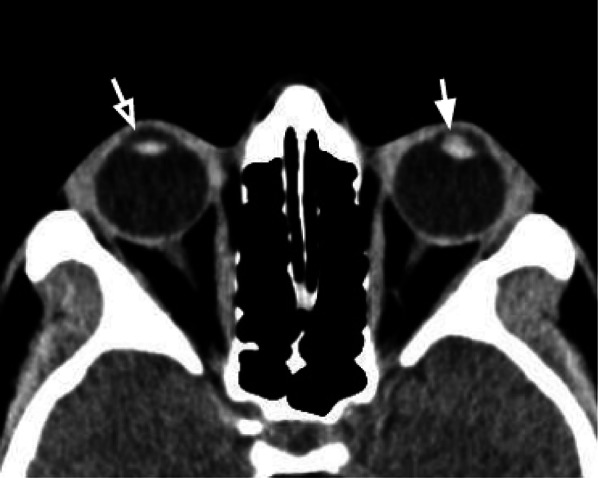
Axial CT acquisition showing a left hyphema, visible as a hyperdense (hemorrhagic) focal component in the anterior chamber, anterior to the lens (white arrow). The content of the contralateral chamber is homogeneous and hypodense (empty arrow)

The anterior chamber is the fluid-filled space, delimited anteriorly by the cornea and posteriorly by the anterior iris surface. Hyphema is a hemorrhage within the anterior chamber of the globe, due to an injury of small blood vessels within the iris and ciliary body, visible on CT as an increased attenuation in the anterior chamber (Fig. 13) [30]. Due to the superficial location, the anterior chamber can be easily assessed clinically, without the need for any imaging; however, the hyphema can limit the ophthalmological evaluation of the posterior chamber for the presence of blood products.

Tips: in the case of hyphema, the role of CT is to assess the posterior chamber and the presence of associated injuries.

### Vitreal hemorrhage

**Fig. 14:**
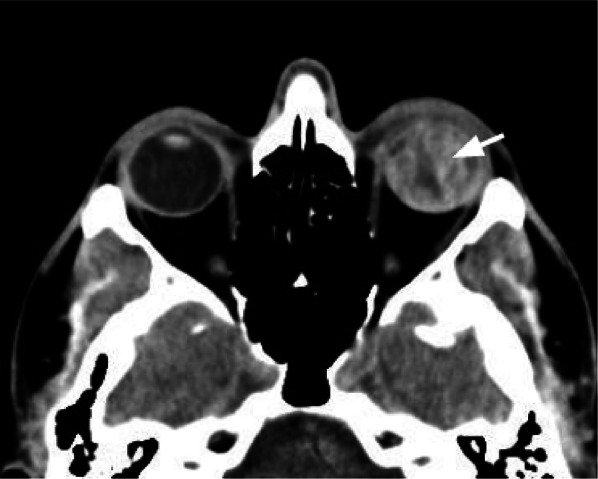
Axial CT acquisition. Left vitreous hemorrhage visible as a diffuse inhomogeneous hyperdensity of the left eyeball (arrow)

The normal vitreous is a high-water-content gel-like fluid, bounded by the posterior and anterior hyaloid membranes, with homogeneous fluid-like density on CT. Its post-traumatic increased density suggests the presence of a vitreous hemorrhage (Fig. 14), which can be a sign of closed-globe, but also of open-globe injury [13, 30]. The coexistence of foreign bodies should be assessed.

Tips: when the vitreal hemorrhage is present, look for the coexistence of foreign bodies and associated orbital injuries.

### Retrobulbar hemorrhage

**Fig. 15:**
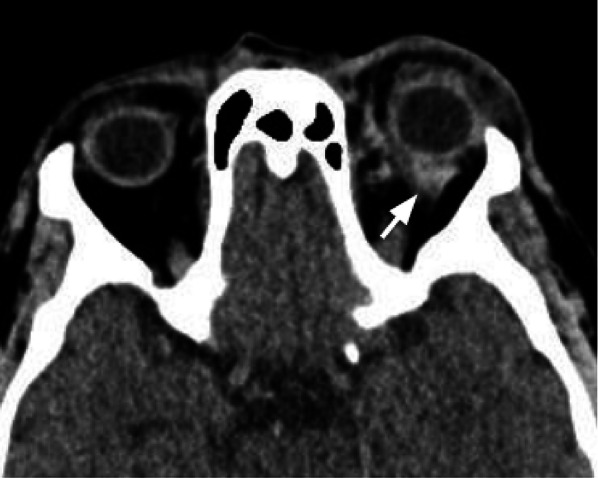
Axial CT acquisition of a retrobulbar hemorrhage visible as a retrobulbar inhomogeneous hyperdensity (white arrow). Diffuse thickening of the left palpebral subcutaneous soft tissues

It consists of an accumulation of blood in the retrobulbar space that causes an increase in the intra-orbital pressure resulting in compression or stretching of the optic nerve and reduced perfusion to the eye (Fig. 15) [13]. On CT, it is visible as hyperdense retrobulbar irregular components.

Tips: look for possible mass effects on the optic nerve.

### Lens detachment

On CT the normal lens is easily recognizable, hyperdense, and well differentiable from the surrounding fluid within the posterior chamber.

**Fig. 16:**
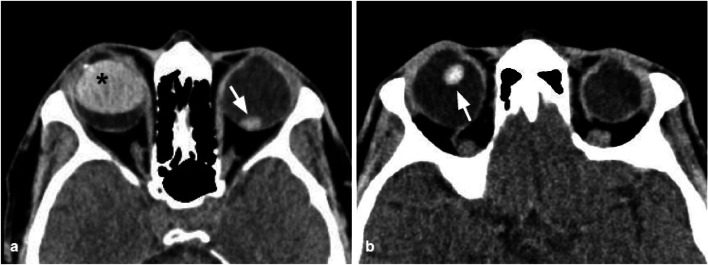
Two cases of post-traumatic lens detachment and dislocation. The left lens (**a**) and the right lens (**b**) are not recognizable in their normal sites and are displaced posteriorly (white arrows). Well-defined hyperdensity related to intraocular silicone oil (black asterisk), due to a previous surgical intervention for a retinal detachment in the right globe in (**a**)

Traumatic deformation of the globe causes the stretch and disruption of the zonular fibers that hold the lens in place, resulting in lens dislocation: subluxation of the lens can either be anterior or posterior, with the latter being more frequent [30]. The diagnosis of lens dislocation is clinical, but CT can help the clinicians through the identification of the lens position and assessment of coexistent injuries (Fig. 16).

Moreover, the lens capsule may be damaged, resulting in a post-traumatic cataract [30, 31]: this finding is visible on CT as a reduced density of the lens due to the fluid accumulation that follows capsular disruption.

Tips: the radiologist should be familiar with the appearance of the lens after surgical treatment of cataracts: the native lens is removed and replaced with a silicone or acrylic lens. The post-surgical lens is thinner than the native one and can be confused in the evaluation of orbital traumas.

### Retinal detachment

The retina is the innermost sensory layer of the globe and consists of an outer layer strictly attached to the choroid, and an innermost layer responsible for visual perception. The layers unite at the ora serrata, which marks the retina's anterior limit and the ciliary body's posterior limit [10].

Retinal detachment is defined as the separation of the sensory retina from the underlying choroid, with the accumulation of subretinal fluid/blood [39].

**Fig. 17:**
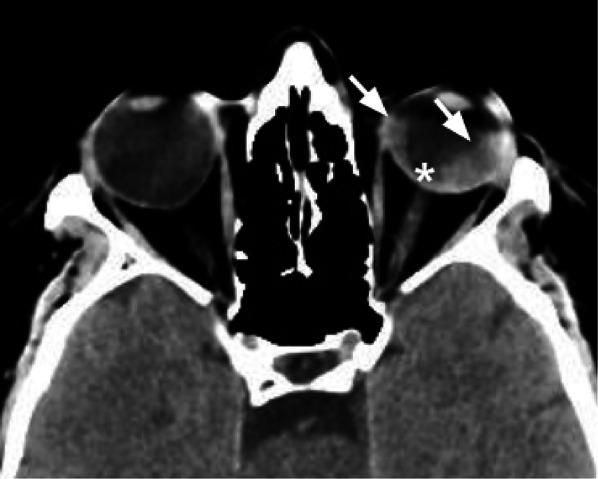
Axial CT acquisition reconstructed with soft tissues algorithm showing a left retinal detachment. Evidence of folded membranes with hyperdense fluid in the subretinal space (white arrows). The detachment converges posteriorly on the optic disc (asterisk)

Different patterns can be visible on CT images, according to the extension of the damage: (I) folded layers with hyperdense fluid in the subretinal space, (II) retinal detachment limited anteriorly by the ora serrata, (III), diffuse retinal detachment, visible as a V-shaped image with the apex at the optic disk (Fig. 17) [30].

Retinal detachments can be treated using scleral bands (pneumatic retinopexy, pars plana vitrectomy, or injection of intraocular silicone oil [39].

### Choroidal detachment

The choroid is the most vascularized tissue in the eye and provides feeding to the outer retinal structures [13, 15]. It runs from the optic nerve head to the ora serrata.

**Fig. 18:**
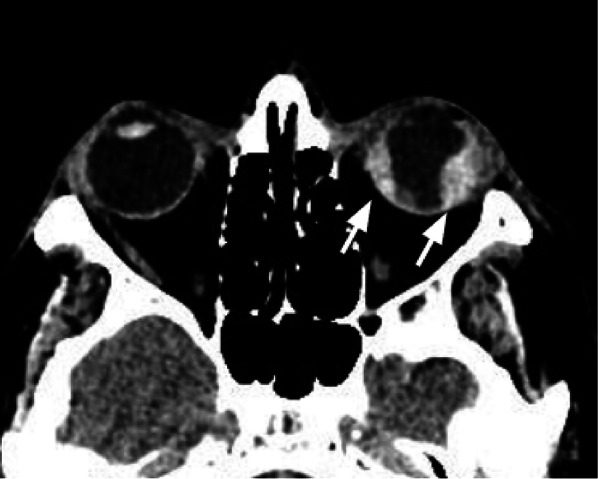
Axial CT acquisition reconstructed with a soft tissue algorithm of a choroidal detachment with choroidal hemorrhage, visible as hyperdense lentiform component on CT (white arrows), that diverges approaching to the optic disc (compared to the retinal detachment that converges to the optic disc)

The choroidal detachment is caused by fluid accumulation in the space between the choroid and the sclera, the suprachoroidal space. It is usually the result of decreased ocular pressure. It is visible on CT as suprachoroidal fluid collections with a biconvex or lentiform shape (Fig. 18), extending from the vortex level to the ora serrata [13, 15].

### Open globe injuries

Open globe injuries are a major cause of blindness. The rupture is most frequent at the insertions of the muscles, where the sclera is thinnest [30].

**Fig. 19:**
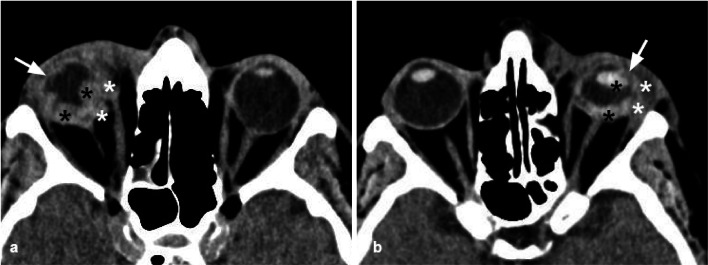
Axial CT acquisition reconstructed with soft tissue algorithm of two examples of post-traumatic open globe injuries of the right (**a**) and left (**b**) eye. The affected globes are dysmorphic and show volume loss, particularly the one in case (**b**). The globe borders are irregular due to scleral interruptions (white asterisks). The anterior contour of the globes is flattened (white arrow). Evidence of irregular vitreous hemorrhagic hyperdensities (black asterisks). Bilateral thickening of the palpebral soft tissues

CT is considered the examination of choice for the assessment of open globe injuries, with a sensitivity > 75% [31]. Findings suggestive of an open globe injury are altered globe contour, globe volume loss (Fig. 19), scleral interruption, intraocular foreign bodies, or intraorbital air.

Tips: Some congenital (coloboma) and acquired (staphyloma) ocular malformations can mimic an open globe injury.

**Fig. 20:**
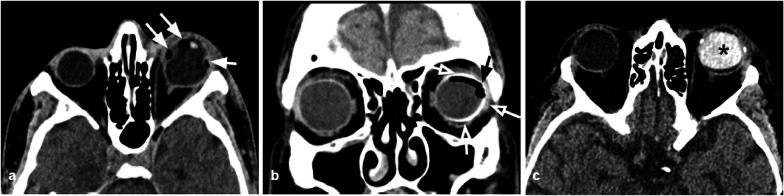
CT appearance of the various ocular interventions that can mimic open globe fractures and/or vitreous hemorrhages. 32-Year-old patients with an increase of the anteroposterior diameter of the left eyeball, related to a myopic eye, previously treated for e retinal detachment, with current CT evidence of intraocular air-bubble-like components (white arrows), corresponding to perfluoropropane gas bubbles (**a**). **b** Coronal CT reconstruction showing a scleral buckle surgery with low attenuation device (black arrow) that mildly indents the temporal aspect of the left globe and encircles the scleral buckle (metallic density ring around the left globe, white empty arrows). **c**. highly attenuation silicone oil in the left globe (asterisk) placed after surgical vitrectomy to treat a retinal detachment. To help the differential diagnosis with hemovitreous, the attenuation values in Hounsfield Units (HU) can be assessed, with silicone oil having values greater than 100 HU

Moreover, the radiologist should be aware of the radiological appearance of the various ocular interventions: previous treatments for retinal detachment and scleral buckle can complicate the diagnosis of an open globe injury. Perfluoropropane gas bubbles (Fig. 20a, b), low-attenuated silicon sponge, and scleral bands can be visible in case of treatment for retinal detachment. Scleral buckle and silicone oil are hyperdense on CT and can be confused with hemorrhage (Fig. 20c). Gas in the globe can be detected post recent pneumatic retinopexy.

### Extraocular muscles damages

Ocular traumas can result in entrapment, avulsion, and laceration of the extraocular muscles. Medial and inferior rectus muscles are mostly involved (Figs. 2, 3, 4, 5). Intramuscular hematoma can worsen the ischemia caused by the entrapment. CT has a high sensitivity in detecting muscle entrapment, while its sensitivity in detecting muscle laceration or intramuscular injury is lower. The contact between the muscles and the fractured edges can increase the risk of diplopia [16].

Tips: Use the coronal reconstruction to better evaluate extraocular muscles and their relationship with the fracture edges.

### Foreign bodies

The identification of intraorbital foreign bodies within the globe is crucial, as retained foreign bodies increase the risk of infectious or inflammatory endophthalmitis [34].

**Fig. 21:**
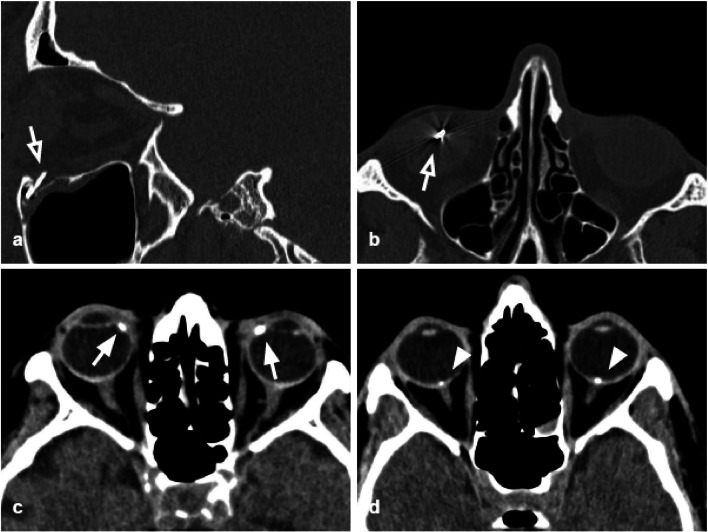
Two examples of radiopaque foreign bodies, with intraorbital (**a**) and intraocular (**b**) location. **a** Sagittal reconstruction with bone algorithm showing an intraorbital hyperdense foreign body (a piece of a glass bottle) embedded in the orbital floor (white empty arrow). **b** Axial acquisition reconstruction with bone algorithm showing an intraocular foreign body causing beam hardening artefacts (metal fragments in a welder, due to an accident at work) (white empty arrow). **c**, **d** Show possible mimickers of foreign bodies: in **c**, bilateral scleral plaques visible as calcifications near the scleral insertion of the extraocular muscles (white arrows); bilateral presence of intraocular lens implants for previous cataract surgery, that are smaller, thinner and less dense compared to the normal lenses. In **d**, bilateral presence of drusen (white arrowheads), visible as focal calcifications in the optic nerve head

CT is usually the first diagnostic tool, as it is extremely sensitive in detecting metallic foreign bodies both in the superficial and deeper structures of the globe as well as soft tissue spaces of the orbit (Fig. 21a, b).

CT also showed high sensitivity in the identification of glass foreign bodies (96% for foreign bodies of 1.5 mm) [40].

The CT sensitivity decreases when dealing with the plastic ones: in these cases, the presence of air within the globe is suggestive for an open-globe injury.

Wooden foreign bodies can present as linear air density images [41].

Indications for surgical removal include reduced visual acuity, mechanical restriction of ocular movements, development of acute or chronic infection, or suppurative reactions. Removal of foreign bodies located close to the apex is also generally discouraged due to the risk of intraprocedural complications.

Tips: wooden foreign bodies can be hypoattenuating and linear and can be mistaken for air: radiologists should be aware of their appearance.

Senile scleral plaques and drusen can also be mistaken for foreign bodies by inexperienced radiologists: it is essential to be aware of these entities (Fig. 21c, d).

### Orbital CT assessment flowchart

**Fig. 22:**
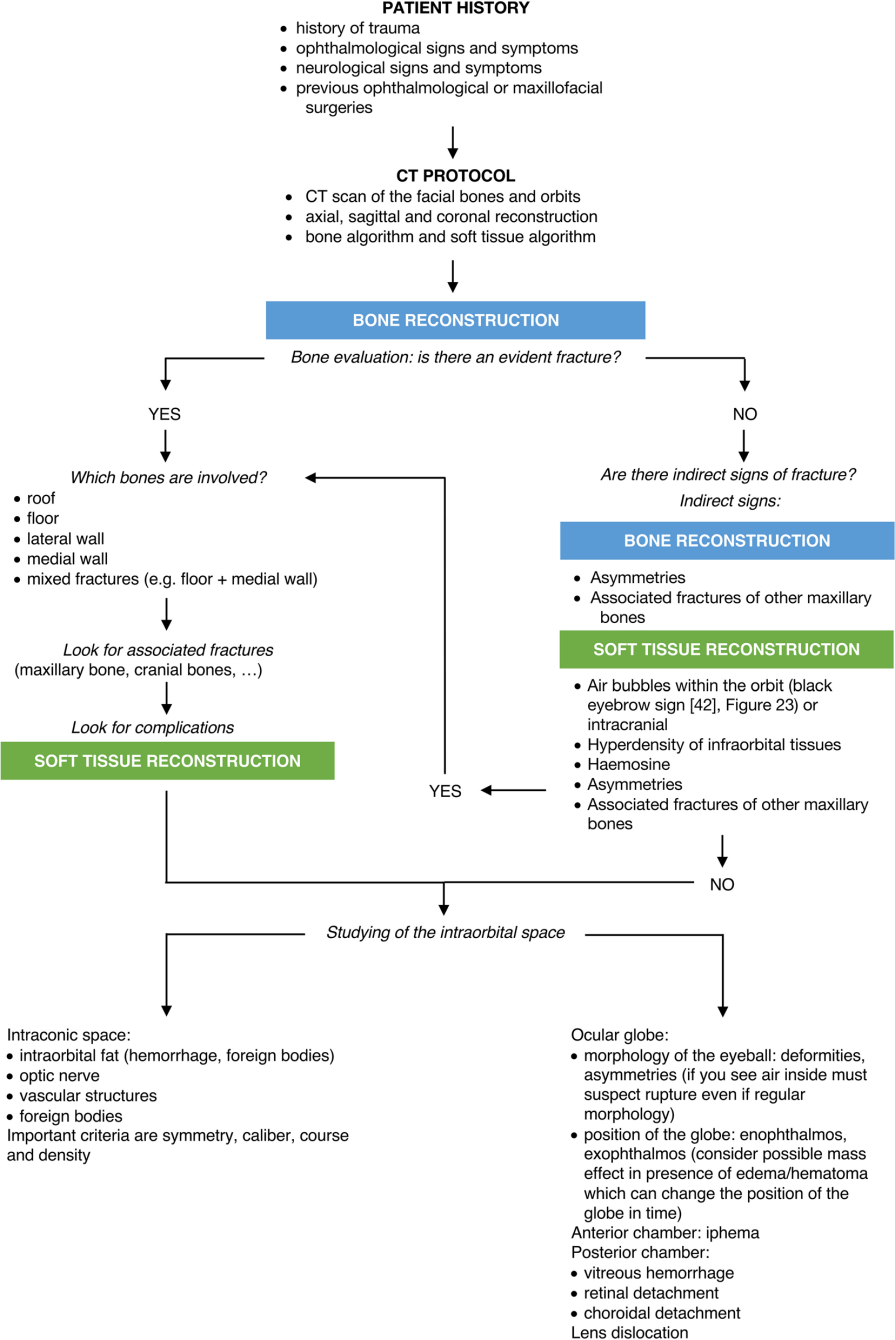
Flowchart showing the radiological approach to the analysis of post-traumatic orbits CT examinations

**Fig. 23:**
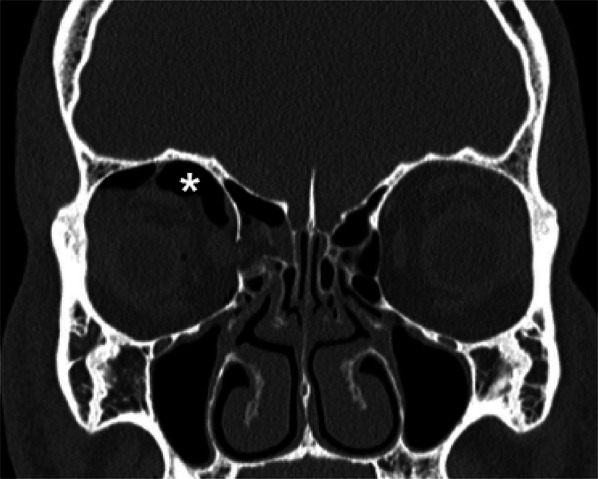
Coronal reconstruction showing the presence of retrobulbar emphysema due to a fracture of the right lamina papyracea, visible as air within the upper right orbit, that mimics the appearance of an eyebrow (asterisk). This is an important indirect sign of orbital bone fracture [42]

We propose a flowchart to help the radiologist in the analysis of maxillofacial CT for orbital traumas (Figs. 22, 23).

## Conclusions

Orbital trauma can occur isolated or in association with craniofacial injuries, resulting in fracture patterns of variable complexity. CT represents the gold standard to assess injuries related to orbital traumas and guides the appropriate management.

Understanding and being familiar with fracture patterns, possible damages to soft tissues, eye and globe abnormalities is crucial for the radiologist for a quick and appropriate diagnosis, and mandatory for the right treatment planning to avoid long-term invalidating consequences.

## Data Availability

The material and data included in the study belong to the Radiology Department of Our Institution and are collected in our Picture archiving and communication system (PACS).

## Abbreviations

BOF: Blow-out fracture
CT: Computed tomography
